# The Early Initiation Advantages of Physical Therapy in Multiple Sclerosis—A Pilot Study

**DOI:** 10.3390/life13071501

**Published:** 2023-07-03

**Authors:** Florin Mihai Marcu, Ilarie Brihan, Anamaria Ciubara, Vasile Valeriu Lupu, Nicoleta Negrut, Anamaria Jurcau, Ancuta Lupu, Stefan Lucian Burlea, Felicia Dragan, Lacramioara Ionela Butnariu, Alina Elena Ignat, Alexandru Bogdan Ciubara

**Affiliations:** 1 Faculty of Medicine and Pharmacy, University of Oradea, 410087 Oradea, Romania; 2 Faculty of Medicine and Pharmacy, “Dunarea de Jos” University of Galati, 800008 Galati, Romania; 3 Faculty of General Medicine, “Grigore T. Popa” University of Medicine and Pharmacy, 700115 Iasi, Romania; 4 Public Health and Management Department, “Grigore T. Popa” University of Medicine and Pharmacy, 700115 Iasi, Romania; 5 “Al. I. Cuza” University, 700506 Iasi, Romania

**Keywords:** multiple sclerosis, physical therapy, central nervous system, quality of life

## Abstract

(1) Background: Multiple sclerosis (MS), a chronic progressive neurological disorder which affects the central nervous system (CNS), can result in disorders of all the functions controlled by the CNS: motor, sensory, cognitive and emotional. Physical therapy (PT), conducted through proprioceptive neuromuscular facilitation (PNF) techniques, can be customized to the individual patient’s needs and has the potential to improve the patient’s evolution. This study aims to establish if PT based on PNF techniques has a beneficial role in MS treatment. (2) Methods: We performed a prospective study on 40 patients who were diagnosed with MS and previously treated only with MS drug treatment (DT). These patients have participated in a PT program throughout one year. At the beginning and at the end of our study, after one year, we have assessed the following parameters: timed walk for 25 feet (Timed 25-Foot Walk test- T25FW test), dexterity of the upper limbs (9-Hole Peg Test—9HPT), disability level (Expanded Disability Status Scale—EDSS) and cognitive function (Paced Auditory Serial Addition Test—PASAT. (3) Results: In subjects in the early stages of MS, lower limb mobility improved significantly, T25FW decreasing from 6.46 to 5.80 (*p* < 0.001) and upper limb ability increased significantly in the dominant hand, 9HPT decreasing from 17.73 to 16.97 (*p* = 0.006) and not significantly in the non-dominant hand, 9HPT decreasing from 17.73 to 17.50 (*p* = 0.255). Furthermore, among these subjects, cognitive performance improved; their PASAT increased from 52.14 to 54.14 (*p* = 0.036), while the level of disability of these subjects improved only slightly, the EDSS scale evolving from 3.08 to 2.91 (*p* = 0.650). (4) Conclusions: In patients with early forms of MS, combining DT with a PT program based on PNF techniques results in: regaining muscle strength in the lower limbs, improving coordination while walking, correcting dexterity in the upper limbs and increasing the ability to concentrate.

## 1. Introduction

Multiple sclerosis (MS) is one of the most common causes of non-traumatic disability in young adults, more common in women [1]. The precise etiology of MS remains unclear [2]. Today its pathophysiological mechanism is considered to correspond, most likely, with an autoimmune disease [3]. Demyelination, specific to this pathology, may coexist with foci of incomplete and dysfunctional remyelination [4]. The result of these morpho-pathological changes is a complex pathology with a wide range of signs and symptoms, including physical, mental and psychiatric disabilities [5].

Depending on the clinical course, MS is known as “the disease of a thousand faces” and has four clinical forms: relapsing-remitting, secondary progressive, primary progressive and progressive relapsing [6,7].

There are therapeutic possibilities, such as physical therapy (PT), that increase the likelihood of changing the natural evolution of the disease, by extending the time between relapses, decreasing the severity of relapse exacerbation and delaying the evolution towards a progressive form [8]. In people with MS (pwMS), a specific physical therapy program (PTP), has a positive influence on: muscle strength, fatigue, joint mobility, spasticity, body weight and mood [9]. Depending on the degree of disability, aerobic PTP slows the progression of the disease and reduces the severity of symptoms [10,11,12].

According to specialized studies, the main types of aerobic exercises that are recommended for a PTP for pwMS are: passive exercises which require the assistance of another person, active and active-resistance exercises, exercises for balance and coordination and exercises and positions for fighting spasticity [13]. The PTP needs to be individualized, flexible and adjusted for the patient’s symptoms, in terms of the intensity, frequency and the duration of the exercises [14]. Early assessment by an experienced MS physiotherapist is recommended to establish an individualized exercise and/or lifestyle physical activity plan [15]. Among pwMS, there may be some symptomatic similarities, but each person has their own clinical image [16].

Proprioceptive neuromuscular facilitation (PNF) is an advanced form of flexibility training. PNF involves both stretching and contracting the targeted muscle to enhance both active and passive range of motions, which optimize motor performance and rehabilitation [17]. A PTP based on the PNF techniques has a direct effect on the motor neurons [18]. Repeated contraction and Hold-Relax-Active movement techniques are used for the hypotonic muscles. Both trigger stretch reflexes and activate the gamma loop, activating alpha motoneurons, and thus, facilitating muscle contraction. For spastic muscles, the rhythmic rotation technique acts on the joint mechanoreceptors, inhibiting the alpha motoneurons of the spastic muscles. Also, the Hold-Relax technique facilitates the Golgi system in the muscle spindle and excites the Renshaw cells in the spinal cord, thereby inhibiting the alpha motoneurons of the spastic muscles [19].

The Multiple Sclerosis Functional Composite (MSFC) test is one of the most used, standardized, multidimensional MS assessment instruments [20]. The three component measures of the MSFC are the Timed 25-Foot Walk test (T25FW), 9-Hole Peg Test (9-HPT) and Paced Auditory Serial Addition Test (PASAT) [21]. In pwMS and people with mild-moderate disabilities with scores of 1.0–5.5 on the Extended Disability Status Scale (EDSS), there are clear benefits of PTP [22]. There are specialized studies that highlight the positive effect of an adapted PTP on memory, learning, attention and the ability to concentrate [23,24].

This study aims to establish the effectiveness of PTP, achieved through PNF techniques, in the treatment of pwMS, who are able to walk and carry out activities daily living (ADL) independently and compliant with drug treatment (DT). We wanted to evaluate whether the initiation of PTP as quickly as possible in pwMS who have been compliant with DT has clear therapeutic benefits.

## 2. Materials and Methods

**Figure 1:**
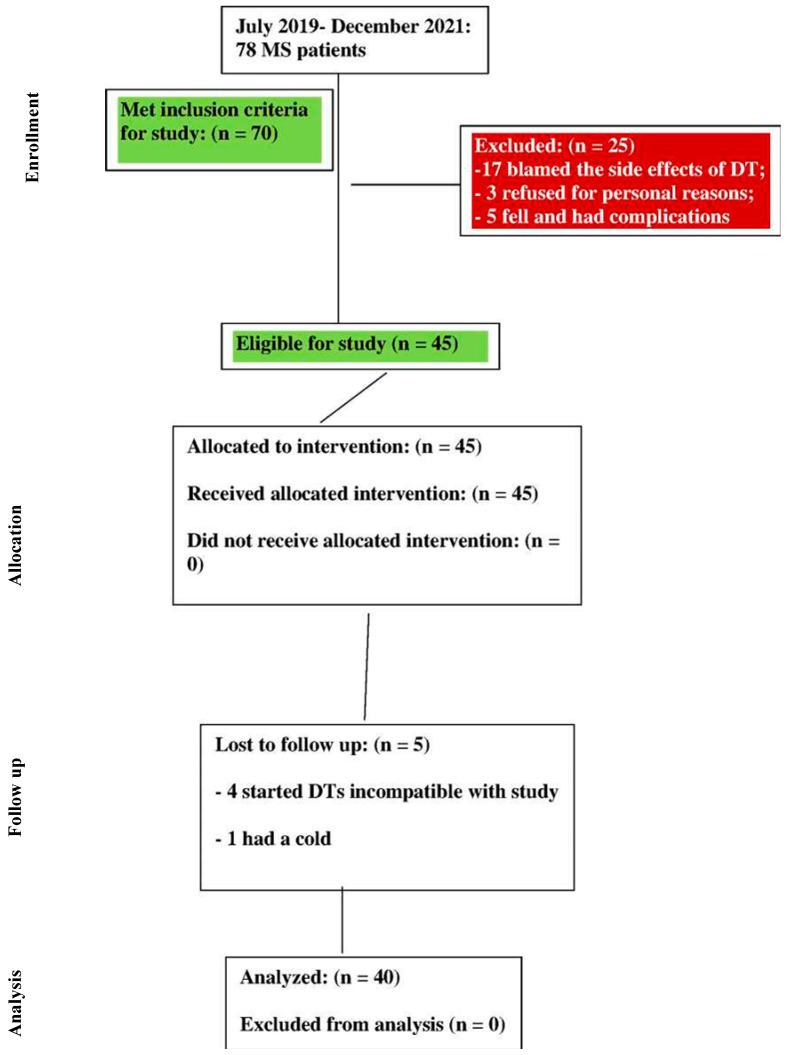
The flow-chart of study.

Forty subjects who have undergone specialized therapy at the Emergency Clinical County Hospital of Oradea, Romania were selected based on subjective and objective anamnesis criteria. During the study period, in the Emergency Clinical County Hospital of Oradea, Romania, there were 78 patients diagnosed with MS. Of these, 70 met the criteria for inclusion in the study. Among these 70 individuals, 20 refused to participate in the study after they left the hospital. Thus, 50 subjects remained who continued the PTP treatment at home. Of these, five subjects fell and could not continue the study due to complications. Another five discontinued the study because they did not perform the PTP sessions three times per week; four of these started treatment with Rivotril and Trittico, and the fifth had a cold (Figure 1). The study took place over the course of more than two years, between July 2019 and December 2021.

Inclusion criteria for entering the clinical study were: confirmed MS diagnosis [25], MS of more than 6 months duration, relapsing-remitting form of MS, written consent for participation, compliance with the DT and PTP throughout the study and compliance with the assessment of the monitored parameters. The DT for each subject was started prior to the study, and all subjects accepted in the study had adapted to each of their DTs.

Exclusion criteria for the clinical study included refusal to participate and alteration of the patient’s general condition throughout the study regardless of the cause. Alterations included neoplasm, debilitating comorbidities, mental illness and any type of complication or disability significant enough to affect the performance of ADL and the subject’s inability to walk independently. To exclude subject selection bias, we respected the potential for natural recovery of MS [26]. Subjects whose medication prevented them from regularly performing weekly PTP were also excluded during the study.

In this study, in order to evaluate the PTP, the evolution of the following parameters was monitored:

1. T25FW test is a quantitative function test to assess lower limb mobility. The score for this test is the average of two attempts. Depending on the severity of the disability, pwMS may use assistive devices when doing this task; assessments were performed by the same physiotherapist [27,28,29].
2. 9HPT is a simple test of fine motor coordination of the upper limbs. The score for each hand is an average of two trials. Two consecutive trials with the dominant hand are immediately followed by two consecutive trials with the non-dominant hand; the assessments were performed by the same physiotherapist [30,31].
3. PASAT is used on a large scale in assessing the cognitive performance of pwMS. The score is the total number correct out of 60 possible answers; the assessments were performed by the same physiotherapist [32,33].
4. EDSS is a method of quantifying disability in MS and monitoring changes in disability levels over time; the assessments were performed by the same neurologist [34,35].

The assessments of the parameters monitored were performed in the morning, around 10 o’clock, before starting the PTP, at time T0 and after one year, when the study ended, at time T1. All study subjects have received DT, initiated prior to the start of our study. DT initiated upon the start of the study was represented by an adjusted and individualized PTP, depending on the patient’s degree of MS and symptoms, throughout the study period. In the hospital, the subjects of the study have undergone PTP for eight weeks, which comprised at least three sessions a week. After this period, each subject was recommended to continue the PTP at home. Home-based PTP of three sesssions per week were monitored by physiotherapists from the Multiple Sclerosis Foundation in the city and student volunteers. Monitoring of subjects at home was carried out as follows: one session per week by home visit and the other two by video call. Each month, subjects who complied with the PTP, were reassessed once in hospital by the same physiotherapist. The PTP was based on a pyramid structure, with basic exercises at the base of the pyramid and more integrated exercises at the top. The pyramid of the PTP progressed through simple exercises to mobilize all the joints used during warm-up and exercises based on PNF techniques. It continued with active mobilization, active-passive, specific strengthening and integrated strength exercises. The top of the pyramid of the PTP included balance and coordination exercises while standing and walking. All exercises in the PTP were aerobic to avoid and delay the onset of fatigue. In the PTP, the principles of gradualness and painlessness were respected. When the patient showed signs of fatigue (sweating, red/white facial coloration, increased breathing rate or the patient was unable to respond to the physiotherapist’s questions), the PTP was interrupted. The PTP started with 2–3 sets, then progressively increased to 4–5 sets with a 2–4 min break between sets, and this was performed three times a week. Between training sessions, a rest period of 24–48 h was respected to avoid fatigue. Each subject performed a 60-min PTP three times in one week, performing a total of 180 min of specific exercises in one week. Thus, 3 h of PTP were performed every week. In total, 24 h of PTP were performed in the hospital and approximately 120 h of PTP were performed at home. Each PTP consisted of 3 parts: warm-up, the actual exercise period and recovery. The warm-up included simple exercises to mobilize all the joints, especially those used during training on that day. Depending on the speed and amplitude of the movement carried out during the warm-up, it could be considered as part of the proprioceptive exercises as the joint receptors could be activated.

The actual exercise period included the following types of aerobic exercises: spasticity control, active mobilization, active with resistance, specific toning, balance and coordination. The recovery period was the period in which the body returns to the cardiovascular parameters before the PTP, and this included exercises similar to the warm-up or walking/biking.

The DT, adjusted depending on the stage of the disease and the patient’s compliance, consisted of treatments:

- that modify the evolution of MS: immunomodulatory drugs, such as Copaxone;
- that modify relapse exacerbation: iv glucocorticoids, such as Solumedrol;
- that address symptoms and rehabilitation: adjusted and individualized as needed, such as vitamins, anti-inflammatory drugs, antidepressants, sedatives.

### Statistical Methods

Statistical data processing was carried out using the SPSS 20 program. Average values of parameters, frequency ranges, standard deviations, tests of statistical significance were calculated using the Student method (test *t*). For comparing averages, ANOVA was used and the level of statistical significance was 0.05. The “sensitivity to change” statistical indicator was also used to assess “Effect size” (ES), which is a measure of the experimental effect [36].

ES is a standardization method for the magnitude of change in a variable after a predetermined period of time. It represents the mean change in a variable expressed as standard deviation units. This standardization allows comparison of values related to the change of a variable in a study. Moreover, ES can be used to compare the same variables between different studies. The calculation formula for ES is: ES = (m1 − m2)/s1; where

m1—mean value of the initial score;

m2—mean value of the score after a determined period of time;

s1—standard deviation value of the initial score.

The interpretation for ES is:

- <0.2—insignificant change
- 0.2–0.49—minor change
- 0.5–0.79—moderate change
- ≥0.8—major change

This study was conducted according to the guidelines of the Declaration of Helsinki and it was approved by the Ethical Commission and the Ethical Council at the Emergency Clinical County Hospital of Oradea, Romania (registration No. 16159/02.VII.2019, 16166/03.VII.2019).

## 3. Results

To describe the subjects enrolled in the study, we included demographics and the interval between diagnosis and initiation of PTP (IDiPTP). The characteristics of the subjects enrolled in the study are presented in Table 1.

The parameters assessed in the study: T25FW test, 9HPT, PASAT and EDSS, were analysed for their differences between study time points, T0—start of the study and T1—end of the study, and these were stratified by the subject’s time interval between diagnosis and initiation of PTP. This interval, IDiPTP, actually represents the duration of the disease at the start of the study from the time of diagnosis establishing. The IDiPTP was divided into three periods:

- IDiPTP < 12 months = early forms of the disease, less than 1 year duration;
- IDiPTP 12–24 months = forms of the disease between 1- and 2-year duration;
- IDiPTP > 24 months = forms of the disease more than 2-year duration.

Thus, the evolution of the subjects throughout the study in terms of the parameters followed in the study, at T0 and T1 and according to the duration of the disease, is shown in Table 2.

As shown in Table 2, subjects with IDiPTP < 12 months have favorable results at the end of the study; i.e., the mobility of the lower limbs of these subjects improved significantly, with the motor coordination of the upper limbs very good on the dominant hand and moderately good on the non-dominant hand. Furthermore, these subjects had a significant improvement in cognitive function. In terms of EDSS, only two of these subjects showed a decrease in EDSS score of 0.5. In contrast, among subjects with IDiPTP between 12–24 months and IDiPTP > 24 months, the evolution is less favorable. It appears, then, that the effect of PTP along with DT decreases in pwMS with longer disease histories. The longer the disease lasts, the less favorable is the evolution of the parameters monitored in the study.

Comparing the EDSS score in terms of the duration of the disease, it is noted that both in subjects with more than 1 year of disease duration and in those with more than 2 years of disease duration the values are close, ranging between 3.5 and 5. In subjects with less than 1 year of disease duration, the EDSS score values are clearly lower, between 2.5 and 4.

In order to highlight the exact number of subjects that improved for each studied parameter, we quantified their evolution in relation to the three disease history strata mentioned above, as can be seen in Table 3.

Subjects who most improved in their clinical evolution during the study are those with an age of disease lower than 1 year. Of these, 5% presented a reduction in the degree of disability, while almost 40% of them walked the 25 steps faster within the T25FW. On the other hand, among the subjects with more than 2 years of disease, only one subject improved in clinical evolution during the study, more precisely, this subject performed the T25FW test faster at the end of the study compared to the beginning of the study.

**Figure 2:**
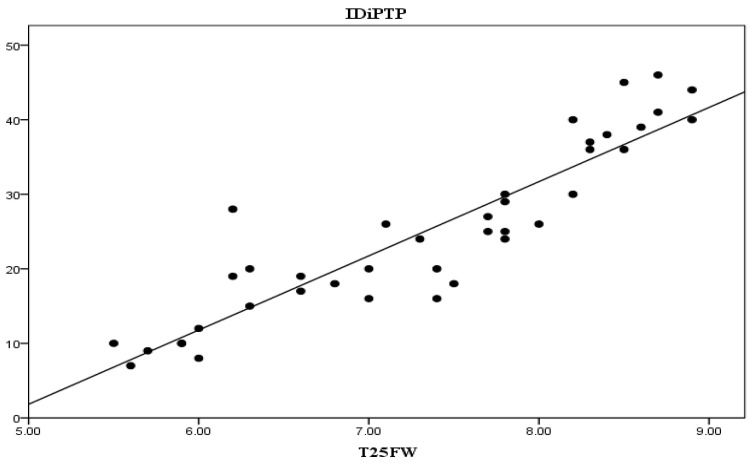
Interval between diagnosis and initiation of PTP—T25FW correlation. Legend: IDiPTP = interval diagnosis—initiation PTP (months), T25FW = Timed 25-Foot Walk (seconds).

**Figure 3:**
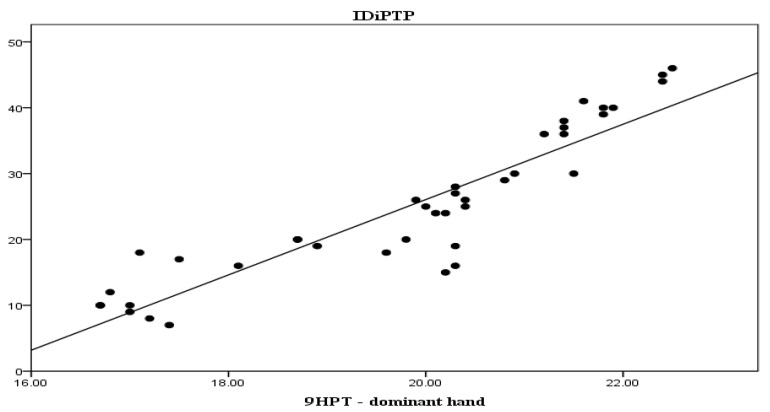
Interval between diagnosis and initiation of PTP—9HPT—dominant hand correlation. Legend: IDiPTP = interval diagnosis—initiation PTP (months), 9HPT = Nine-Hole Peg Test (seconds).

**Figure 4:**
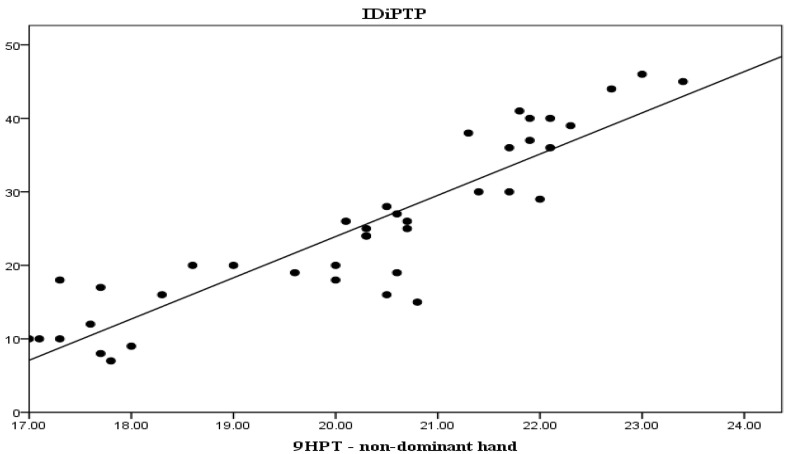
Interval between diagnosis and initiation of PTP—9HPT—non-dominant hand correlation. Legend: IDiPTP = interval diagnosis—initiation PTP (months), 9HPT = Nine-Hole Peg Test (seconds).

In order to highlight the relationship between the duration of the disease and the present symptomatology we aimed to establish how changes in the three modifiable parameters of the study, i.e., T25FW test, 9HPT and PASAT, relate to the IDiPTP of subjects. Thus, we established correlations between IDiPTP and change in T25FW test results (r = 0.844, *p* < 0.001), seen in Figure 2, and between IDiPTP with changes in 9HPT, dominant hand and non-dominant hand (r = 0.813, *p* < 0.001 and r = 0.80, *p* < 0.001, respectively), seen in Figure 3 and Figure 4.

From the three graphs presented above, it is clear that there is a direct proportional relationship between IDiPTP and the T25FW, 9HPT scores.

**Figure 5:**
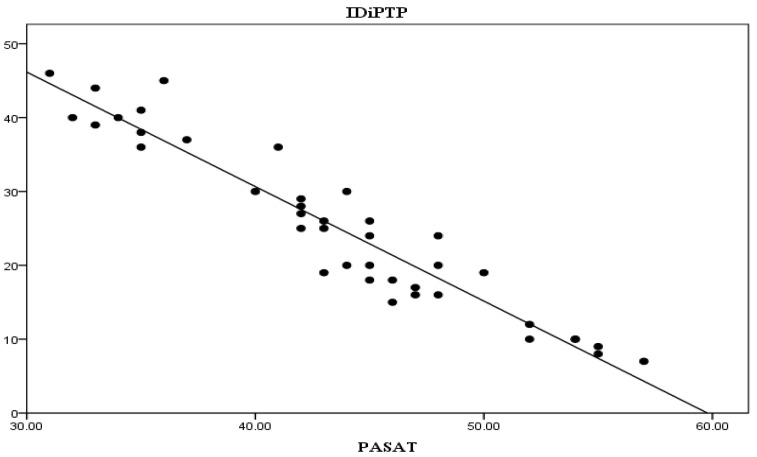
Interval between diagnosis and initiation of PTP—PASAT correlation. Legend: IDiPTP = interval diagnosis—initiation PTP (months), PASAT = Paced Auditory Serial Addition Test (points).

We also established an inverse correlation with the PASAT score (r = 0.899, *p* < 0.001), see Figure 5.

These correlations highlight that the longer the interval between diagnosis and PT initiation, the greater the improvement in the T25FW and 9HPT scores are, while the PASAT score is inversely, increasingly lower.

## 4. Discussion

Fatigue is the most common symptom in MS and its presence has a clear impact on the ability of the patient rehabilitation, as it decreases the capacity to improve both physical and cognitive activities. [37]. Although fatigue may be independent of depression and cognitive dysfunction, these three conditions often occur simultaneously and interact to increase the extent of disability [38]. A graded aerobic PTP with appropriate rest periods and avoidance of overtraining is recommended [39]. According to expert studies, aerobic exercise improves patient’s endurance and coordination. These benefits are extremely important in the PT programs for pwMS.

In our study the subject’s endurance and coordination were assessed in terms of walk, and it was noted that one subject walked 7.62 min slower at time T1 than at time T0. However, the clear benefits of an aerobic PTP based on PNF techniques on walking are noticed mostly in subjects who have an IDiPTP not more than 12 months. This aspect emphasizes the fact that PTP benefits in terms of stability and coordination of walking are more obvious when MS is in an incipient form, less serious, with fewer deficiencies established. According to the results of our study, a PTP based on PNF techniques, coupled with a suitable medication treatment, for a duration of 12 months, leads to an improvement in the T25FW test. These results are in alignment with data in the literature [40,41] and they underline the positive effects of PTP on stability, muscle strength and walk of patients with MS. People with MS expend more energy when walking compared to controls. The longer the duration of the disease is, the greater this energy consumption is and, obviously, the benefits of PTP are smaller. This increase in energy consumption contributes to the development of fatigue. Thus, the results of our study agree with studies by Stella and Roney [42,43].

In the early functional stages of MS, the intensity and frequency of kinetic exercises must be adapted so that pwMS do not experience fatigue, this is an imperative aspect of the RP [44]. There are times when they will have to ease up, but pwMS discover their own limits and learn to listen to the signals sent by their own body [45]. As the disease progresses, fatigue sets in faster and faster in pwMS, so with more advanced disease, pwMS can perform less intense PTPs [46]. Fatigue is a common and profound barrier to intensive PT, and it reduces the benefits of PT [47].

One major symptom of MS in the hands is a loss of dexterity, as fine-motor skills decrease or disappear. MS patients have difficulty with maintaining grip and activities such as picking things up, using writing utensils or buttons on clothes and controlling cutlery [48]. Through comparative evaluation of the results obtained at T1 and T0, we noted the positive impact of PTP has had on the ability of pwMS with an incipient form of the disease.

As MS progresses, the ability to concentrate and perform fine movements are increasingly affected so that the fine motor ability is reduced and PTP benefits decrease. This aspect is also underlined by the results of the study which showed that, in pwMS with three years of disease and without prior PT, the results of 9HPT tests at time T1 are lower in comparison with time T0.

Moreover, in terms of PTP impact on the upper limbs, benefits for the dominant hand are higher in comparison those of the non-dominant hand. This issue is also confirmed by the results obtained throughout other similar studies [49,50].

Another very important aspect of PTP is the improvement of mood with consequent reduction of depression and cognitive impairment [51]. In performing specific kinetic exercises, it is very important that the physiotherapist sequences the exercises and teaches the patient to focus on the correctness of the exercises. PTPs decrease stress and help pwMS to be more relaxed, aspects that clearly increase the mood of pwMS [52].

In pwMS, PTP is associated with modest improvements of attention, memory and cognitive ability. The further MS progresses, the more obvious cognitive impairments are. There is a direct link between performing PT at a certain intensity and the patient’s mood. As the patient’s ability to perform PT decreases, depression, one of the most frequent symptoms of MS, worsens and cognitive ability declines [53]. In pwMS, changes in both fatigue and depressive symptoms are significantly and directly associated with residual changes in physical activity [54]. The negative impact of depression on cognitive function also represents an argument to explain why the results of the PASAT test at T1 are lower than those recorded at T0 [55]. Favorable results of the PASAT test are noted only in subjects who are in an incipient form of the pathology, who have the pathology for less than 1 year, and these results correlated with other studies [56,57].

In terms of disability assessed using EDSS, a minor improvement is observed in subjects with a disease duration less than 1 year. Lower EDSS values were found in those pwMS in an early phase of the disease who still have a relatively good capacity to perform physical effort. This shows, as in other studies, a clear correlation between the level of disability, the ability to exercise and the initial stage of the disease [58,59]. With longer MS progression are higher EDSS scores and reduced ability to perform physical therapy [60]. In these pwMS, a PTP only has the role of slowing down the worsening of the disability, to maintain their independence [61].

The results of our study, confirmed by other peer-reviewed studies [62,63,64], reflect that, among patients with early forms of MS who are able to walk and perform ADLs independently, starting PTP as early as possible, in combination with DT, has consecutive benefits from the cognitive and disability perspectives.

## 5. Conclusions

In the group with IDiPTP < 12 months, the changes in parameters studied here were significantly better than among those with IDiPTP between 12–24 months or >24 months. The results of our study reflect the advantages of PTP initiation as soon as possible, performed using PNF techniques, in pwMS with early forms who already adhere to DT. In these cases, we recorded the following benefits:

(i) body stability and coordination during the gait;
(ii) upper limb ability;
(iii) cognitive function and ability to concentrate.

## Figures and Tables

**Table 1:** Initial characteristics of the batch enrolled in study.

Batch Characteristics	m.v. ± SD	min–max	No.	%
Age (years)	33.53 ± 4.42	25–41		
Sex—F/M			26/14	65/35
BG—U/R			29/11	72.5/27.5
IDiPTP (months)	25.00 ± 11.35	7–46		

Legend: F = female sex, B = male sex, BG = background, U = urban, R = rural, IDiPTP = diagnosis-initiation of PTP interval, m.v. = mean value, SD = standard deviation, min.-max. = minimum and maximum value, No. = number of subjects, % = percentage values.

**Table 2:** Progression of parameters throughout the study depending on the duration of MS.

Progression	T0	T1	*p*	ES
IDiPTP < 12 months				
T25FW test	6.46 ± 0.29	5.80 ± 0.20	<0.001	2.28
9HPT—dominant hand	17.43 ± 0.26	16.97 ± 0.26	0.006	1.78
9HPT—non-dominant hand	17.73 ± 0.34	17.50 ± 0.37	0.255	0.67
PASAT	52.14 ± 1.35	54.14 ± 1.77	0.036	1.49
EDSS	3.08 ± 1.47	2.91 ± 1.62	0.650	0.21
IDiPTP 12–24 months				
T25FW test	7.35 ± 0.37	6.94 ± 0.52	0.028	1.13
9HPT—dominant hand	19.32 ± 0.61	19.19 ± 0.68	0.613	0.21
9HPT—non-dominant hand	19.48 ± 0.68	19.41 ± 0.72	0.743	0.13
PASAT	46.92 ± 1.89	46.31 ± 1.93	0.420	0.33
EDSS	4.05 ± 1.97	4.05 ± 1.97	1	0
IDiPTP > 24 months				
T25FW test	8.23 ± 0.47	8.12 ± 0.65	0.526	0.24
9HPT—dominant hand	21.06 ± 0.85	21.22 ± 0.81	0.546	0.18
9HPT—non-dominant hand	21.39 ± 0.94	21.69 ± 0.91	0.457	0.13
PASAT	40.11 ± 4.01	38.88 ± 4.65	0.416	0.31
EDSS	4.18 ± 2.23	4.18 ± 2.23	1	0

Legend: T25FW = Timed 25-Foot Walk, 9HPT = Nine-Hole Peg Test, PASAT = Paced Auditory Serial Addition Test, EDSS = Expanded Disability Status Scale, T0 = initial time, T1 = final time, *p* = statistical significance, ES = effect size, IDiPTP= interval diagnosis—initiation PTP.

**Table 3:** The number of patients with clinically relevant amelioration for each parameter.

Parameters	T25FW Test	9HPT Dominant Hand	9HPT Non-Dominant Hand	PASAT	EDSS
IDiPTP < 12 months	15	7	3	8	2
IDiPTP 12–24 months	7	2	1	0	0
IDiPTP > 24 months	1	0	0	0	0

## Data Availability

The data is available on request from the corresponding author.
